# Increasing cure rates of solid tumors by immune checkpoint inhibitors

**DOI:** 10.1186/s40164-023-00372-8

**Published:** 2023-01-16

**Authors:** Weijie Ma, Ruobing Xue, Zheng Zhu, Hizra Farrukh, Wenru Song, Tianhong Li, Lei Zheng, Chong-xian Pan

**Affiliations:** 1 Chinese American Hematologist and Oncologist Network, New York, NY USA; 2 Department of Pathology and Laboratory Medicine, Dartmouth-Hitchcock Medical Center, Lebanon, NH 03756 USA; 3 Ellis Fischel Cancer Center, University of Missouri, 1 Hospital Dr, Columbia, MO 65201 USA; 4 Department of Medicine, Brigham and Women’s Hospital, Boston, MA USA; 5 Kira Pharmaceuticals, Cambridge, MA USA; 6 Department of Medicine, Division of Hematology & Oncology, University of California Davis, Sacramento, CA 95817 USA; 7 The Sydney Kimmel Comprehensive Cancer Center, Johns Hopkins University School of Medicine, Baltimore, MD 21287 USA; 8 VA Boston Healthcare System, Boston, MA 02132 USA; 9 Department of Medicine, Brigham and Women’s Hospital, Harvard Medical School, Boston, MA USA; 10 Department of Medicine, VA Northern California Health Care System, Mather, CA, USA

**Keywords:** Immunotherapy, Immune checkpoint inhibitor, Neoadjuvant therapy, Adjuvant therapy, Lung cancer, Gastrointestinal cancers, Genitourinary cancers, Breast cancer, Head and neck cancer, Melanoma, Gynecological cancer

## Abstract

Immunotherapy has become the central pillar of cancer therapy. Immune checkpoint inhibitors (ICIs), a major category of tumor immunotherapy, reactivate preexisting anticancer immunity. Initially, ICIs were approved only for advanced and metastatic cancers in the salvage setting after or concurrent with chemotherapy at a response rate of around 20–30% with a few exceptions. With significant progress over the decade, advances in immunotherapy have led to numerous clinical trials investigating ICIs as neoadjuvant and/or adjuvant therapies for resectable solid tumors. The promising results of these trials have led to the United States Food and Drug Administration (FDA) approvals of ICIs as neoadjuvant or adjuvant therapies for non-small cell lung cancer, melanoma, triple-negative breast cancer, and bladder cancer, and the list continues to grow. This therapy represents a paradigm shift in cancer treatment, as many early-stage cancer patients could be cured with the introduction of immunotherapy in the early stages of cancer. Therefore, this topic became one of the main themes at the 2021 China Cancer Immunotherapy Workshop co-organized by the Chinese American Hematologist and Oncologist Network, the China National Medical Products Administration and the Tsinghua University School of Medicine. This review article summarizes the current landscape of ICI-based immunotherapy, emphasizing the new clinical developments of ICIs as curative neoadjuvant and adjuvant therapies for early-stage disease.

## Introduction

**Fig. 1:**
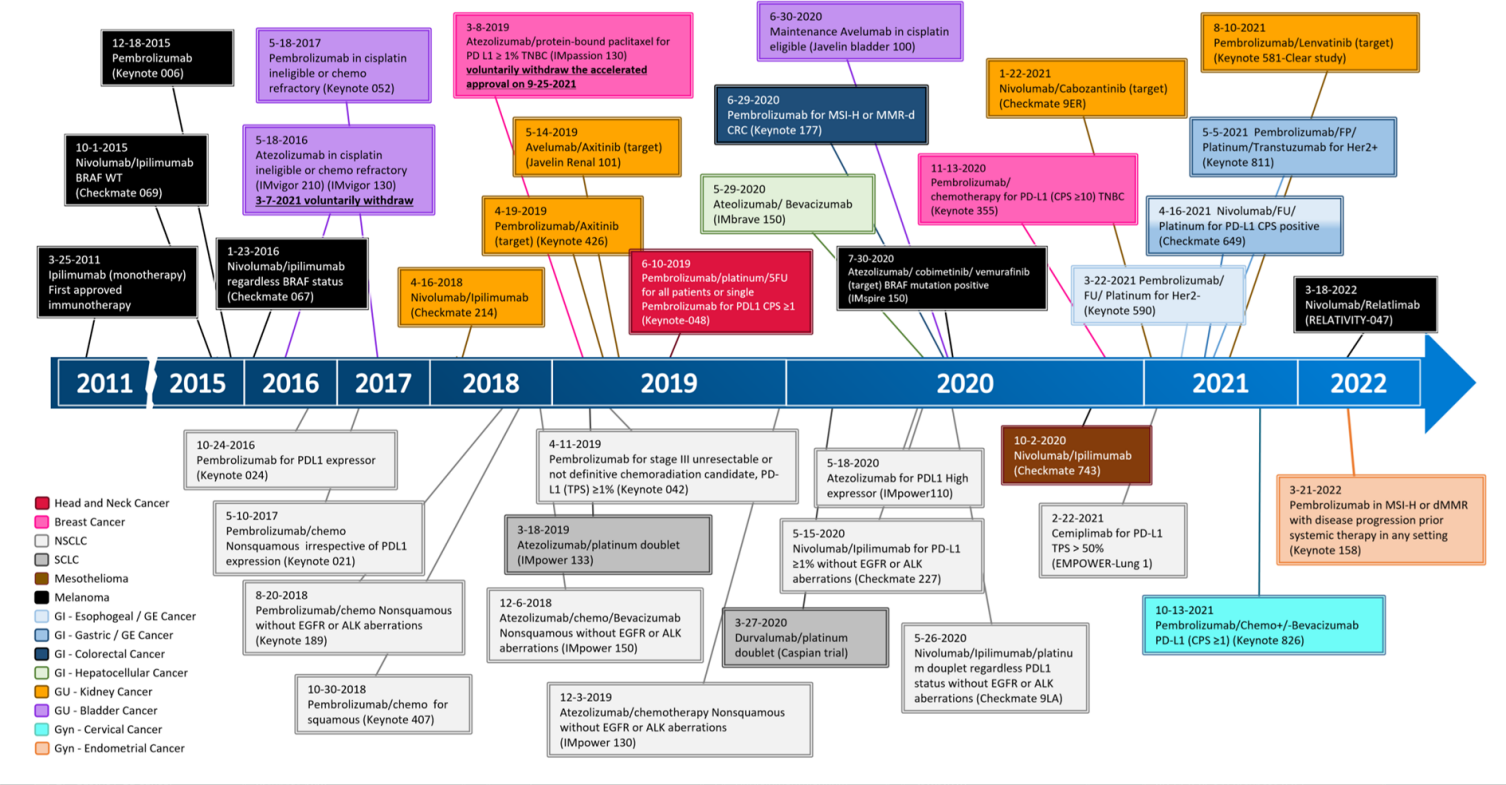
FDA approvals of first-line immunotherapy for advanced/metastatic cancer

**Fig. 2:**
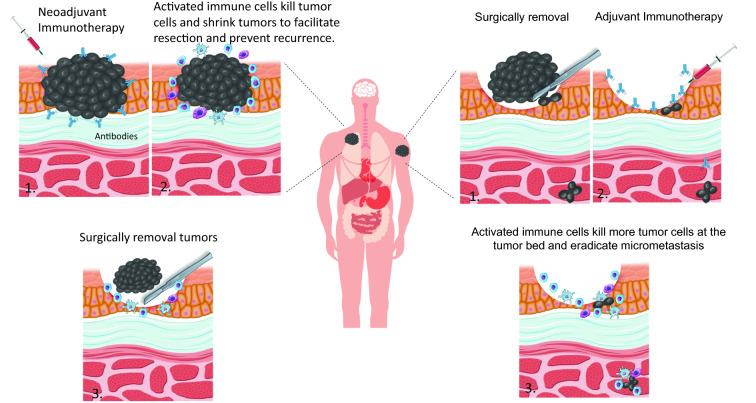
Neoadjuvant vs Adjuvant Immunotherapy in solid tumors

**Fig. 3:**
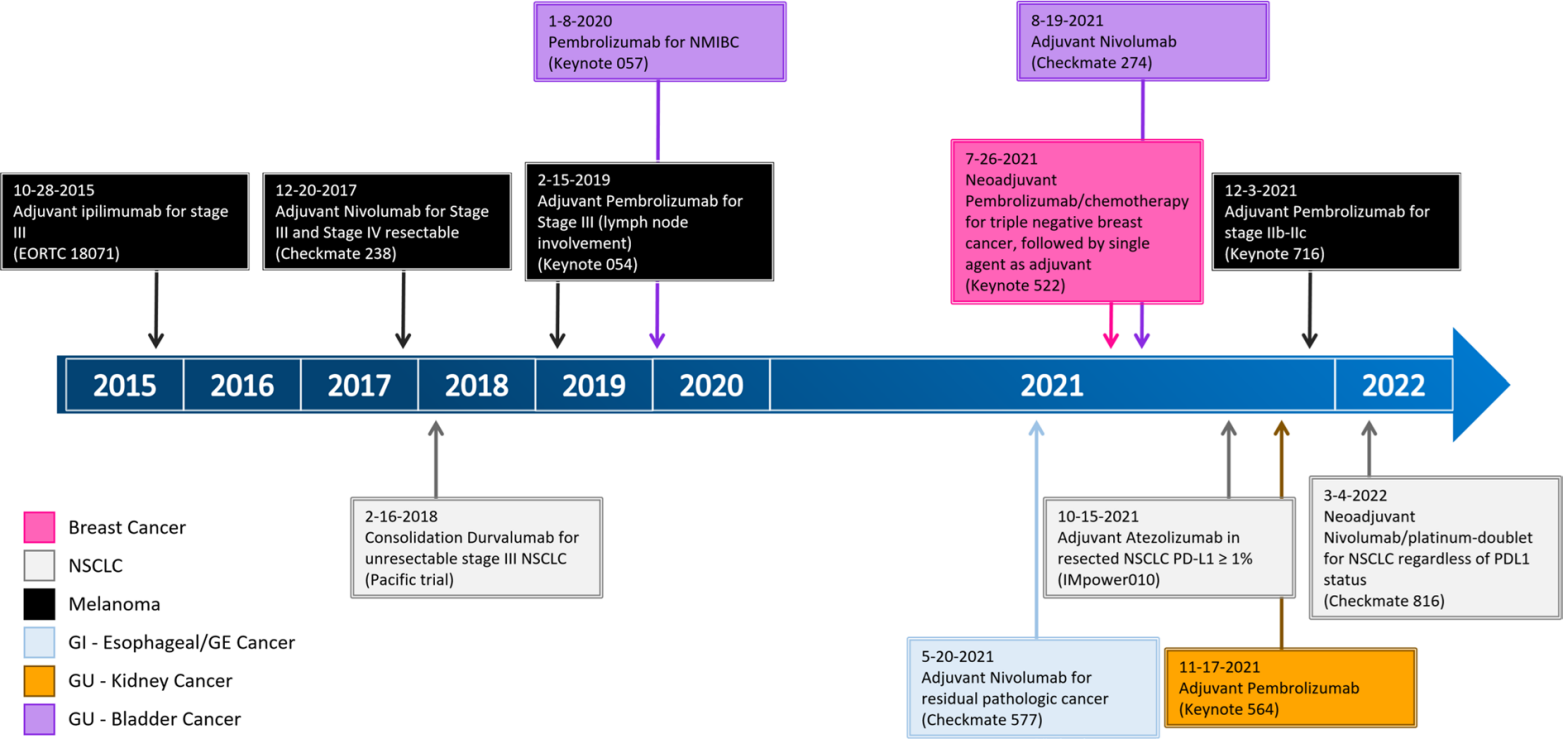
FDA approvals of neoadjuvant and adjuvant immunotherapy for localized cancer

The past decade has witnessed the emergence of systemic and personalized therapies in cancer treatment. After the approval of the first immune checkpoint inhibitor (ICI), ipilimumab, targeting cytotoxic T-lymphocyte antigen-4 (CTLA-4), initial investigations with ICIs were focused on salvage therapy for metastatic cancers. As a salvage therapy, ICIs have demonstrated objective responses that, compared with conventional chemotherapy, are often durable. Gradually, ICIs have heralded a new era in treating advanced and metastatic cancers, moving from the last resort to first-line therapy (Fig. 1). Historically, immunotherapy, including many cancer vaccines, was often tested in localized cancer in the adjuvant setting following the completion of conventional adjuvant therapy. In the neoadjuvant setting, immunotherapy, including ICIs, was studied for its immuno-biological effects on resectable cancers (Fig. 2) [1–3]. It was then discovered how a single treatment of ICIs can lead to radiographic responses at an equivalent rate to chemotherapy and, subsequently, complete pathological responses at least at a similar rate as chemotherapy in some resected cancers [4, 5]. This result, coupled with the evidence of durable responses of ICIs in metastatic cancers, has increased the interest in examining the role of ICI therapy in patients with early-stage cancers. ICIs have been tested in numerous clinical trials for their curative intent and have already become standard of care in the neoadjuvant and/or adjuvant therapies for melanoma, non-small cell lung cancer (NSCLC), bladder cancer, and breast cancer (Fig. 3).

This review article summarizes the recent developments in cancer immunotherapy with ICIs, including those discussed at the 2021 China Cancer Immunotherapy Workshop. This annual workshop has been co-organized by the Chinese American Hematologist and Oncologist Network (CAHON), the China National Medical Products Administration (NMPA) and Tsinghua University since 2015 [6–9]. Throughout this review, we will integrate the recent developments in cancer immunotherapy, discuss strategies for treating advanced stages with a noncurative intent and explore the new paradigm of applying immunotherapy in the neoadjuvant and adjuvant setting with curative intent in localized tumors for major cancers.

### Lung cancer

There are major developments for immunotherapy in both small cell lung cancer (SCLC) and NSCLC. In NSCLC, immunotherapy has been approved as neoadjuvant therapy and maintenance therapy for nonmetastatic disease, in addition to its use as a single agent or combination first-line therapy for metastatic NSCLC. At the 2021 China Cancer Immunotherapy Workshop, Patrick Forde, MD from Johns Hopkins University, presented a comprehensive review on the development of neoadjuvant immunotherapy in lung cancer.

#### Non-small cell lung cancer (NSCLC)

NSCLC has witnessed the paradigm shift of ICIs from salvage therapy for metastatic cancers to neoadjuvant/adjuvant therapy for early-stage cancers. For patients with advanced NSCLC, first-line systemic treatment generally consists of targeted therapy, immunotherapy, cytotoxic chemotherapy, or a chemo-immunotherapy combination depending on the tumor's expression of programmed death-ligand 1 (PD-L1), histology type (squamous versus nonsquamous) and the presence of driver mutations, such as epidermal growth factor receptor (EGFR) activation mutation and anaplastic lymphoma kinase (ALK) translocation. In case there is no specific driver mutation, ICIs are commonly used as single agents for those NSCLCs with PD-L1 expression of over 50% and with little cancer-related symptoms. So far, three ICIs have been approved by the United States (U.S.) FDA as single agents for the treatment of advanced NSCLC: two anti-PD1 (Programmed Cell Death Protein 1) antibodies, pembrolizumab based on the KEYNOTE-024 trial [10, 11] and cemiplimab based on the EMPOWER-Lung 1 trial [12], and one anti-PD-L1 antibody atezolimab based on the IMpower 110 trial [13]. In cases when PD-L1 expression is less than 50% or in patients with symptomatic cancer or high disease burden, ICI-based combination is commonly used. Currently, the nivolumab plus ipilimumab combination demonstrated superior survival compared with chemotherapy in the CheckMate-227 trial, leading to its approval for metastatic NSCLC (squamous or non-squamous) without EGFR or ALK genomic alterations with PD-L1 expression  ≥ 1% [14, 15]. The nivolumab and ipilimumab combination is also approved in metastatic NSCLC (squamous or non-squamous) after two cycles of platinum-doublet chemotherapy based on the CheckMate-9LA trial [16]. In addition, for non-squamous NSCLC, platinum/pemetrexed plus pembrolizumab (based on the KEYNOTE-189 trial) [17, 18], carboplatin/nabpaclitaxel plus atezolizumab (based on the IMpower130 trial) [19], and carboplatin/paclitaxel plus atezolizumab and bevacizumab (based on the IMpower150 trial) were also FDA approved as a first-line therapy [20]. For squamous NSCLC, pembrolizumab plus carboplatin with either paclitaxel or nabpaclitaxel has been approved for advanced squamous NSCLC following a phase III KEYNOTE 407 trial with 559 patients [21].

##### Durvalumab as consolidation therapy after chemoradiation therapy for unresectable stage III NSCLC

Despite multimodality treatment, the outcome of unresectable stage III NSCLC remains poor, with a five-year survival rate of approximately only 15% [22]. As ICIs have dramatically altered the therapeutic landscape in advanced NSCLC, a randomized phase III PACIFIC trial was conducted to move one step back and determine the efficacy of an anti-DP-L1 antibody durvalumab in Stage III NSCLC. This trial included 700 patients with unresectable stage III NSCLC who did not have disease progression after at least two cycles of platinum-based chemoradiation [23, 24]. Patients were randomized to durvalumab or placebo for up to 12 months at a 2:1 ratio. Durvalumab significantly improved the overall survival (OS) (hazard ratio or HR 0.68; 99.73% CI 0.47–0.997; p = 0.0025) with the 24-month OS rate of 66.3% with durvalumab maintenance compared to 55.6% of the placebo control arm (p = 0.005). Longer follow-up revealed that the median survival in the group treated with durvalumab was 47.5 months, compared to 29.1 months in the placebo control (HR 0.72; 95% CI 0.59–0.89) and 5-year OS was 42.9% versus 33.4% in the control [25]. Subgroup analyses suggest that the survival benefit of durvalumab was not observed in the group with the PD-L1 expression of less than 1% [26]. For the first time, ICI-based immunotherapy demonstrated a benefit for localized NSCLC.

##### Neoadjuvant ICI-based therapy for resectable NSCLC

The paradigm change for NSCLC is to administer immunotherapy for resectable tumors with curative intent. The CheckMate 816 trial evaluated neoadjuvant therapy with nivolumab plus platinum-doublet chemotherapy in 358 patients newly diagnosed with resectable stage IB to IIIA NSCLC. No sensitizing EGFR or ALK mutations were allowed. Patients were stratified by cancer stage, PD-L1 status, and sex. Patients were randomly assigned 2:1 to receive nivolumab at 360 mg every three weeks plus chemotherapy for three cycles vs. the same chemotherapy schedule. Then, patients underwent radiological staging and surgery within six weeks of neoadjuvant therapy. They had the option of adjuvant therapy with or without radiation therapy. An exploratory arm of nivolumab plus ipilimumab was closed early. The primary endpoint was pathological complete response (pCR) by blinded independent review, defined as no viable residual tumor in the resected primary tumor and lymph nodes after surgery. Neoadjuvant nivolumab plus platinum-doublet chemotherapy significantly improved pCR rates compared with chemotherapy alone (24% vs. 2.2%; p < 0.0001). The magnitude of pCR benefit with nivolumab was similar between patients with stage IB and IIIA disease with squamous and nonsquamous histologies, regardless of PD-L1 status and tumor mutational burden. The major pathological response rate among patients who underwent surgery was 46.8% in the nivolumab-containing arm vs. 12.7% in the chemotherapy alone arm. The radiographic objective response rate based on scans was 54% with nivolumab plus chemotherapy vs. 37% with chemotherapy alone. At a minimum follow-up of 21 months, the coprimary endpoint of median event-free survival (EFS) was 31.6 months for the nivolumab plus chemotherapy group and 20.8 months for the chemotherapy alone group. This corresponded to a significant reduction in the risk for disease progression, recurrence, or death of 37% in favor of nivolumab plus chemotherapy (HR 0.63; 95% CI: 0.45–0.87; p = 0.0052).

More recently, the Phase II NEOpredict-Lung trial was presented at the European Society for Medical Oncology (ESMO) Congress 2022. In this randomized, multicentric Phase II study, 60 patients with NSCLC stage IB to IIIA were randomized to 4 weeks of anti-PD1 antibody nivolumab (arm A) or nivolumab plus anti-LAG-3 (Lymphocyte activation gene-3) antibody relatlimab (arm B) before curative surgery. R0 resection was performed in 98% of patients. Radiological response rates were 11% (arm A) and 27% (arm B) per RECIST and complete or major histopathological response rates were 28% (arm A) and 32% (arm B), suggesting further clinical trials of this combination are warranted [27].

##### Adjuvant atezolizumab therapy for stage IIB-III NSCLC with PD-L1 ≥ 1%

IMpower010 was a phase III study evaluating adjuvant atezolizumab (1200 mg every 21 days; for 16 cycles or 1 year) or best supportive care (observation and regular scans for disease recurrence) after adjuvant platinum-based chemotherapy (one to four cycles) in resected stage IB–IIIA NSCLC. After a median follow-up of 32.2 months, atezolizumab treatment improved disease-free survival compared with best supportive care in 495 patients in the stage II–IIIA population whose tumors expressed PD-L1 on  ≥ 1% of tumor cells (HR 0.66; 95% CI 0.50–0.88; p = 0.0039) and in all patients in the stage II–IIIA population (HR 0.79; 0.64–0.96; p = 0.020). In the intention-to-treat population, the HR for disease-free survival was 0.81 (0.67–0.99; p = 0.040). IMpower010 showed a disease-free survival benefit with atezolizumab versus best supportive care after adjuvant chemotherapy in patients with resected stage II–IIIA NSCLC, with pronounced benefit in the subgroup whose tumors expressed PD-L1 on 1% or more of the tumor cells and no new safety issues. Based on this trial, the US FDA approved one year of atezolizumab therapy after adjuvant chemotherapy for stage II-III NSCLC with PD-L1 > 1% [28].

As both neoadjuvant and adjuvant ICIs are available for resectable stage II-IIIA NSCLC, further study is needed to compare these two approaches to determine which yields better clinical benefit. Ongoing phase III studies will determine the role of adjuvant ICIs in patients who received neoadjuvant ICI-chemotherapy (Table 1).

**Table 1:** Ongoing Phase 3 neoadjuvant chemoimmunotherapy trials in solid tumors

Organ system	Clinical trial	Cancer type	Phase	Participant numbers	Trial design	Estimated study completion (year)	Primary endpoint(s)
Lung	NCT02998528 (CheckMate 816)	Early Stage NSCLC	III	350	Nivolumab Plus Ipilimumab or Nivolumab Plus Platinum Doublet Chemotherapy VS Platinum Doublet Chemotherapy	2028	Event-Free Survival (EFS); Pathological Complete Response (pCR)
	NCT03456063 (IMpower030)	Resectable Stage II, IIIA, or Select IIIB NSCLC	III	453	Atezolizumab or Placebo in Combination With Platinum-Based Chemotherapy	2026	Event Free Survival
	NCT04025879 (CA209-77 T)	Surgically Removable Early Stage NSCLC	III	452	Neoadjuvant Chemotherapy Plus Nivolumab VS Neoadjuvant Chemotherapy Plus Placebo, Followed by Surgical Resection and Adjuvant Treatment With Nivolumab or Placebo	2024	Event-Free Survival
	NCT03425643 (KEYNOTE-671)	Resectable Stage II, IIIA, and Resectable IIIB (T3-4N2) NSCLC	III	786	Platinum Doublet Chemotherapy ± Pembrolizumab (MK-3475) as Neoadjuvant/Adjuvant Therapy	2026	Event Free Survival; Overall Survival
	NCT03800134 (AEGEAN)	Resectable Stages II and III NSCLC	III	800	Neoadjuvant/Adjuvant Durvalumab for the Treatment of Patients	2024	Event-Free Survival; Pathological Complete Response
	NCT05157776	Resectable Locally Advanced NSCLC Harboring no Driver Mutations	III	72	Neoadjuvant Sintilimab and Platinum-based Chemotherapy	2023	Pathologically complete response rate
GI tract	NCT04807673	Esophageal Squamous Cell Carcinoma	III	342	Pembrolizumab Plus Paclitaxel and Cisplatin VS Neoadjuvant Chemoradiotherapy Followed by Surgery	2028	Event Free Survival
	NCT04848753	Resectable Locally Advanced Thoracic Esophageal Squamous Cell Carcinoma	III	500	Perioperative Toripalimab (JS001) Combined With Neoadjuvant Chemotherapy	2026	Event Free Survival
	NCT04973306	Esophageal Squamous Cell Carcinoma	II-III	176	Anti-PD-1 antibody (Tislelizumab, BeiGene) combined with neoadjuvant chemoradiotherapy VS neoadjuvant chemoradiotherapy followed by minimally invasive esophagectomy	2027	Major pathologic response; OS
	NCT04304209	Colorectal Cancer	II-III	195	Neoadjuvant Sintilimab ± Chemoradiotherapy	2026	Pathologic complete response rate
	NCT02743494 (CheckMate 577)	Resected stage II-III esophageal or GE junction cancer	III	794	Neoadjuvant chemoRT followed by complete resection, with residual disease adjuvant Nivolumab VS placebo	2025	Disease Free Survival
	NCT05270824	Advance Gastric Adenocarcinoma	III	120	Radical surgery after neoadjuvant immunotherapy (albumin Paclitaxel + Seggio + PD-1 inhibitor) VS adical surgery after neoadjuvant chemotherapy (albumin Paclitaxel + Seggio)	2027	CD8 + tumor-infiltrating lymphocytes in tumor tissue
	NCT04882241	Gastric Cancer	III	120	Pembrolizumab (MK-3475) Plus Chemotherapy (XP or FP) VS Placebo Plus Chemotherapy (XP or FP) as Neoadjuvant/Adjuvant Treatment	2025	Event-Free Survival (EFS); Pathological Complete Response (pCR);Overall Survival
GU	NCT03732677	Muscle Invasive Bladder Cancer	III	988	Durvalumab in Combination With Gemcitabine + Cisplatin for Neoadjuvant Treatment Followed by Durvalumab Alone	2026	Event-Free Survival; Pathological Complete Response
	NCT04700124	Muscle Invasive Bladder Cancer	III	784	Perioperative Enfortumab Vedotin Plus Pembrolizumab (MK-3475) VS Neoadjuvant Gemcitabine and Cisplatin	2026	Event-Free Survival; Pathological Complete Response
	NCT04209114	Muscle-Invasive Bladder Cancer (MIBC) Who Are Cisplatin Ineligible	III	540	Neoadjuvant and Adjuvant Nivolumab Plus NKTR-214, VS Nivolumab Alone Versus Standard of Care	2024	Event-Free Survival; Pathological Complete Response
	NCT04209114	MIBC ineligible for cisplatin	III	540	Neoadjuvant and Adjuvant Nivolumab + Bempeg/NKTR-214, VS Nivolumab Alone vs Standard of Care w radical cystectomy	2024	Event-Free Survival; Pathological Complete Response
	NCT04700124 (KEYNOTE-B15)	Cisplatin-eligible Muscle Invasive Bladder Cancer	III	784	Perioperative Enfortumab Vedotin + Pembrolizumab 1 yr VS Neoadjuvant Gemcitabine and Cisplatin	2026	Event-Free Survival; Pathological Complete Response
GYN	NCT03038100	Newly-Diagnosed Stage III or Stage IV Ovarian, Fallopian Tube, or Primary Peritoneal Cancer	III	1301	Paclitaxel, carboplatin and atezolizumab for 6 cycles and bevacizumab VS paclitaxel, carboplatin and placebo	2023	PFS, OS
Head and Neck	NCT03700905	Head and Neck Cancer	III	276	Nivolumab Alone or in Combination With Ipilimumab as Immunotherapy VS Standard Follow-up	2024	Disease Free Survival
	NCT03765918 (Keynote 689)	Stage III-IVA Resectable Locoregionally Advanced Head and Neck Squamous Cell Carcinoma	III	704	Pembrolizumab as Neoadjuvant Therapy and in Combination With Standard of Care as Adjuvant Therapy	2026	Major Pathological Response (mPR);Event-free Survival
	NCT05125055	Oral Squamous Cell Carcinoma	II-III	80	Neoadjuvant Toripalimab and Albumin Paclitaxel /Cisplatin VS Docetaxel/ Cisplatin/ 5-fluorouracil (TPF) on Pathological Response in Patients	2025	Major pathologic response
	NCT04557020	High-risk Nasopharyngeal Carcinoma	III	200	Toripalimab with neoadjuvant cis Platinum and gemcitabine VS Standard cis Platinum and gemcitabine	2024	PFS
Skin	NCT04949113	Macroscopic Stage III Melanoma	III	420	Neoadjuvant Ipilimumab Plus Nivolumab VS Standard Adjuvant Nivolumab in Macroscopic Stage III Melanoma	2027	Event Free Survival
	NCT04949113 (NADINA)	Stage III Melanoma	III	420	Neoadjuvant Ipilimumab + Nivolumab (adjuvant Nivo in residual disease or dabrafenib/tremetinib in BRAF V600 mut) VS Standard Adjuvant Nivolumab 1 yr	2027	Event Free Survival
Breast	NCT03725059 (KEYNOTE-756)	Early-Stage Estrogen Receptor-Positive, Human Epidermal Growth Factor Receptor 2-Negative (ER + /HER2-) Breast Cancer	III	1240	Pembrolizumab VS Placebo in Combination With Neoadjuvant Chemotherapy and Adjuvant Endocrine Therapy	2031	Event-Free Survival; Pathological Complete Response
	NCT03036488	Triple Negative Breast Neoplasms	III	1174	Pembrolizumab Plus Chemotherapy VS Placebo Plus Chemotherapy as Neoadjuvant Therapy and Pembrolizumab vs Placebo as Adjuvant Therapy	2025	Event-Free Survival; Pathological Complete Response
	NCT04613674	Triple Negative Breast Cancer	III	581	Camrelizumab Plus Chemotherapy VS Placebo Plus Chemotherapy as Neoadjuvant Therapy i	2023	Pathological complete response rate
	NCT02620280 (NeoTRIPaPDL1)	TNBC	III	278	Neoadjuvant Atezolizumab or placebo with Carbo/abraxane, surgery followed by adjuvant chemotherapy	2022	Event Free Survival
	NCT03726879 (IMpassion050)	Early Her2 + Breast Cancer	III	454	Neoadjuvant Atezolizumab or Placebo wtih Neoadjuvant ddAC Followed By THP, adjuvant Atezo 1 yr VS placebo with Her2 HP/TDM1	2023	Pathological Complete Response
	NCT03595592 (APTneo)	Her2 + breast cancer	III	650	Neoadjuvant chemotherapy VS Atezolizumab + AC-TCHP VS Atezolizumab + TCHP, surgery followed by adjuvant Atezo + HP	2026	Event Free Survival
	NCT04109066 (Checkmate 7FL)	High-risk, ER + , HER2-Early Breast Cancer	III	1200	Nivolumab VS Placebo in Combination With Neoadjuvant Chemotherapy and Adjuvant Endocrine Therapy	2032	Pathological Complete Response, Event Free Survival
	NCT03281954	TNBC	III	1520	Neoadjuvant Chemotherapy With Atezolizumab or Placebo Followed by Adjuvant Atezolizumab or Placebo	2024	Pathological Complete Response, Event Free Survival

HNSCC headneck squamous cell carcinom, NSCLC non-small-cell lung cancer, TNBC triple-negative breast cancer, OS overall survival, PFS progression free survival, VS versus

#### Small cell lung cancer (SCLC)

SCLC is a poorly differentiated neuroendocrine tumor, representing approximately 15% of lung cancers. Even though the Tumor, Node, Metastasis (TNM) staging classification is recommended, patients are often divided into limited-stage (LS) versus extensive-stage (ES) disease. LS-SCLC is limited to the ipsilateral hemithorax and regional lymph nodes and can be confined to one safe radiotherapy field. ES-SCLC has spread beyond this and often has distant metastases.

For LS-SCLC, surgery for stage I and radiation for stages II and III, supplemented with systemic chemotherapy, are preferred. Prophylactic cranial irradiation (PCI) following chemotherapy is the standard of care for patients who achieve a complete or good partial response following treatment. It has been shown to increase OS in patients with LS-SCLC. No ICI, however, has been approved for LS-SCLC to date.

For ES-SCLC without symptomatic brain metastasis, the first-line therapy is platinum-etoposide chemotherapy paired with an anti-PD-L1 antibody, followed by maintenance immunotherapy with an ICI until progression. Two humanized monoclonal anti- PD-L1 antibodies, atezolizumab and durvalumab, have been approved for the treatment of ES-SCLC in combination with etoposide-platinum for induction and maintenance therapy based on the Impower133 and CASPIAN trials, respectively [29, 30]. Even though OS was significantly improved with addition of an ICI to the first-line chemotherapy, there was no significant improvement (less than 2.5%) of the CR rate compared to the control. Hence, this addition is less likely to induce “cure”.

### Breast cancer

Treatment options and recommendations are very personalized in breast cancer and depend on several factors. The decision for upfront therapy is not only determined by the stage, age and menopausal status, but is also guided by the genomic markers and expression status of hormonal receptor and human epidermal growth receptor 2 (HER2). Currently, ICIs have been approved for triple-negative breast cancer (TNBC). Before starting treatment for TNBC, genomic testing for germline breast cancer genes (BRCA) and PD-L1 expression should be conducted. For symptomatic or rapidly progressive TNBC, patients should be treated with combination therapy to achieve a fast and higher response rate. For TNBC in the absence of symptoms or rapidly progressive disease, sequential single-agent chemotherapy is preferable, and the addition of an ICI is preferable in cases of positive PD-L1 expression. To date, the combination of pembrolizumab and chemotherapy has gained FDA approval for patients with locally recurrent, inoperable or metastatic TNBC with a PD-L1 combined positive score (CPS)  ≥ 10 based on the KEYNOTE-355 trial [31].

In order to improve the cure rate of early-stage TNBC, several clinical trials have been conducted to determine the efficacy of the addition of an ICI to neoadjuvant chemotherapy in TNBC [32–34]. Only pembrolizumab has been approved by the FDA. In the randomized, double-blinded Phase III KEYNOTE 552 trial [32], 1174 patients were randomized at a 2:1 ratio to chemotherapy plus pembrolizumab or chemotherapy plus placebo. Chemotherapy included 4 3-week cycles of weekly paclitaxel plus carboplatin (weekly or every three weeks) followed by four additional 3-week cycles of doxorubicin or epirubicin plus cyclophosphamide. During chemotherapy, patients received pembrolizumab 200 mg or placebo once every three weeks. After surgery, patients received pembrolizumab or placebo once every three weeks for nine more cycles. The addition of pembrolizumab significantly improved the pCR from 51.2% to 64.8%, with an estimated treatment difference of 13.6% (95% CI 5.4–21.8%, p < 0.001), and both the PD-L1-positive and -negative groups benefited [32]. In the second interim analysis, the addition of pembrolizumab significantly improved the 36-month event-free survival from 77% of the placebo group to 85% (HR 0.63; 95% CI 0.48–0.82; p < 0.001) [35]. As pCR correlates with OS in patients with early-stage breast cancer [36], adding pembrolizumab to chemotherapy improves survival when combined with ICI or chemotherapy alone.

### Genitourinary cancers

Among the major genitourinary malignancies, prostate and testicular cancers have few mutations and are usually considered minimally immunogenic and less responsive to immunotherapy with ICIs. At the 2021 China Cancer Immunotherapy Workshop, Chong-xian Pan, MD, PhD, MS, from Harvard Medical School reviewed the recent advances in immunotherapy for bladder and kidney cancers.

#### Bladder cancer

Bladder cancer has a high tumor mutation burden and, hence, is considered to be more immunogenic. From 2016 to 2017, five ICIs were approved for the treatment of advanced bladder cancer for carboplatin-ineligible patients or as salvage therapy after disease progression within 12 months of platinum-containing chemotherapy [37–43], even though some of these accelerated approvals were subsequently withdrawn [44]. The unprecedented benefits of immunotherapy in advanced malignancy have increased interest in exploiting these immune stimulatory agents in earlier stages.

##### Non-muscle-invasive bladder cancer (NMIBC)

The standard of care for NMIBC in high-risk patients is transurethral resection of bladder tumor (TURBT) followed by intravesical instillation of a therapeutic agent, usually Bacillus Calmette-Guerin (BCG). This treatment, however, is associated with a 10-year cancer recurrence of 74% and cancer progression can be seen in up to one-third of patients [45]. Upon disease progression, pembrolizumab was approved for patients with BCG-unresponsive, high-risk NMIBC who were not eligible for or declined cystectomy based on KEYNOTE-057. BCG-unresponsive NMIBC is commonly defined as stage progression at three months, persistent high-risk NMIBC at six months, or recurrent high-risk NMIBC within nine months after adequate BCG treatment. The study was a single-arm phase II trial with 96 patients and a median follow-up of 36 months, which demonstrated a 41% (39/96) three-month CR rate. However, the durable response of 12 months or longer was only 19% (18/96) [46].

##### Locally advanced muscle-invasive bladder cancer

For locally advanced bladder cancer, neoadjuvant cisplatin-based chemotherapy followed by radical cystectomy, with its associated urinary diversion, is the standard of care [5, 47]. For cisplatin-ineligible patients, replacement of cisplatin with carboplatin is usually not preferred, and upfront radical cystectomy should be performed, as several studies suggest that carboplatin may be inferior to cisplatin [48, 49]. For patients who receive initial treatment with definitive surgery and are at high risk for recurrence [50], adjuvant nivolumab is indicated [51] based on the CheckMate-274 trial. This was a randomized, double-blind, placebo-controlled, multicenter trial that evaluated 709 patients with urothelial carcinoma originating in the urinary bladder or upper urinary tract who were at high risk of recurrence [51]. The pathological staging criteria were ypT2-ypT4a or ypN + after neoadjuvant chemotherapy and pT3-pT4a or pN + for patients who did not receive neoadjuvant chemotherapy. Adjuvant nivolumab for up to one year significantly improved the mean disease-free survival to 20.8 months from 10.8 months with placebo (HR: 0.70; 98.22% CI, 0.55–0.90; p < 0.001).

Many neoadjuvant immunotherapy-based clinical trials are currently underway for this disease group, either as a single agent, such as atezolizumab in the ABACUS trial [52] and pembrolizumab in the PURE-01 trial [4], or as combination therapy, such the durvalumab/tremelimumab [53] and nivolumab/ipilimumab in the NABUCCO trial [54]. These phase I/II trials have indicated a comparable pathological CR (30–40%) to neoadjuvant therapy [5]. Although these studies demonstrate promising preliminary results, no FDA approvals have been granted in a neoadjuvant setting.

#### Kidney cancer

Kidney cancer is another highly immunogenic genitourinary cancer. No other cancer has more first-line immunotherapy combinations approved than kidney tumor. Historically, before ICIs, interleukin-2 (IL-2) and interferon were used to treat renal cancer and melanoma. IL-2 is highly toxic with most patients requiring critical care, and an objective response is observed in approximately 15% of patients, with some achieving long-term remission [55]. The response to interferon is usually not durable. Hence, these two agents are rarely used nowadays. So far, five ICI-based combinations have been approved by the FDA (Table 2): nivolumab and ipilimumab [56–58], pembrolizumab and axitinib [59, 60], avelumab and axitinib [61, 62], nivolumab and cabozantinib [63], and pembrolizumab plus lenvatinib [64]. All five immunotherapy combinations significantly improved PFS and, in some cases, OS compared with the standard-of-care, sunitinib. A combination of lenvatinib plus pembrolizumab demonstrated the highest objective response rate of 71.0%, although it was also associated with the highest severe adverse event rate and discontinuation of therapy secondary to toxicity [64]. Hence, the treatment decision is mainly based on the physician’s preference, disease status, treatment schedule, underlying medical condition and insurance coverage.

**Table 2:** Clinical trials of first-line therapies for metastatic kidney cancer

Trials	CheckMate 214 Nivolumab + Ipilimumab IV q3w X 4 then q4W	Keynote 426 Pembrolizumab + Axitinib 5–10 mg po bid	Javelin 101 Avelumab + Axitinib	CheckMate 9ER Nivolumab + Cabozantinib 40 mg po qd	CLEAR Pembrolizumab + Lenvatinib 20 mg po qd
Number of Patients	1096	861	886	651	1069 (3 arms)
Primary endpoints	ORR, PFS and OS int/poor	OS and PFS	OS and PFS in PD-L1 +	PFS	PFS, OS and safety
ORR% (vs sunitinib %)CR% (vs sunitinib %)	42 (vs 27) CR: 9 (vs 1)	59.3 (vs 35.7) CR: 5.8 (vs 1.9)	51.4 (vs 25.5) CR: 4.4 (vs 2.1)	56 (vs 27) CR: 8 (vs 5)	71.0 (vs 53.3%) (Len + evero) vs 36.1% (sunitinib) 16.1% vs 9.8% vs 4.2%
OS hazard ratio	0.66 (0.53–0.82)	0.53 (0.38–0.74)	0.78 (0.55–1.08)	0.60 (0.40–0.89)	0.66 (0.49–0.88)
PFS (months) vs sunitinib	12.4 vs 12.3 HR: 0.98 (0.79–1.23)	15.1 vs 11.1 HR: 0.69 (0.57–0.84)	13.8 vs 8.0 HR: 0.69 (0.56–0.83)	16.6 vs 8.3 HR: 0.51 (0.41–0.64)	23.9 vs 14.7 vs 9.2 HR: 0.39
Grade 3 and 4 toxicity % (sunitinib %)	46 (vs 63)	62.9 (vs 58)	71.2 (vs 71.5)	61 (vs 51)	82.4 vs 83.1 vs 71.8
Toxicity-induced discontinuation % (sunitinib %)	22 (vs 12)	10.7 (vs 13.9)	7.6 (vs 13.4)		37.2 (vs 14.4)
FDA approval	04/16/2018	04/22/2019	05/14/2019	01/21/2021	08/11/2021

ORR objective response rate, PFS progression-free survival, OS overall survival, CR complete response, vs versus

For localized kidney cancer, definitive surgery resection is curative and, therefore, the preferred treatment for patients with stage I and II disease (limited to the kidney), stage III disease (extending into major veins or perinephric tissues and/or regional lymph nodes) and even in patients with limited metastasis. No neoadjuvant therapy is currently indicated.

The U.S. FDA has approved adjuvant therapy with pembrolizumab for patients at intermediate-high to high risk of cancer recurrence following nephrectomy, or after nephrectomy and resection of oligometastatic lesions. This was based on the double-blinded placebo-controlled phase III KEYNOTE-564 trial. Pembrolizumab improved the 24-month DFS compared with placebo in the entire study population (77.3 versus 68.1%, HR 0.68; 95% CI 0.53–0.87; p = 0.002). The estimated 24-month OS of 96.6% versus 93.5% of the placebo (HR: 0.54; 95% CI: 0.30–0.96) also showed statistical significance, but long-term follow-up is in progress [65]. All patients with pT2 tumors with grade 4 or sarcomatoid features, pT3 and high-grade tumors, and metastasectomy with no evidence of disease are candidates to undergo adjuvant therapy post-nephrectomy.

Even though adjuvant pembrolizumab therapy improves clinical outcomes, two other adjuvant trials failed to show clinical benefit. In the IMmotion010 trial with atezolizumab, 778 patients were randomized to one year of adjuvant atezolizumab or placebo treatment after nephrectomy. At a median follow-up of 44.7 months, no significant improvement of investigator-assessed disease-free survival was observed: 57.2 months for atezolizumab versus 49.5 months in the placebo group (HR 0.93, 95% CI 0.75–1.15, p = 0.50) [66]. Similarly, the adjuvant nivolumab and ipilimumab combination did not improve disease-free survival over the placebo control in the CheckMate 914 trial: HR, 0.92; 95% CI, 0.71–1.19; P = 0.5347 [67].

### Melanoma

The first ICI therapy approved by the FDA for cancer treatment is ipilimumab for advanced melanoma in 2011, which opened a new chapter of cancer therapy. During the early era of immunotherapy with ICIs, several phase III clinical trials showed that the single agent ipilimumab significantly improves OS [68, 69]. However, with the development of more active and less toxic anti-PD-1 therapies, ipilimumab has become less favorable clinically and is rarely, if ever, used as a single agent in clinics. Currently, for both BRAF-mutant and BRAF-wild-type melanoma, ICI combination therapy has become the preferred first-line therapy for metastatic and recurrent melanoma. Even though targeted therapy, including the combination of BRAF and MEK inhibitors, can yield a rapid response in melanoma with BRAF V600-activating mutations, almost all patients treated with targeted therapy develop resistance. In contrast, the response to ICI therapy can be long-lasting. Furthermore, the response rate of immunotherapy after disease progression on targeted therapy tends to be lower compared with that of an ICI when used as the upfront therapy [70], while there is no significant difference in the response rate whether targeted therapy is used upfront or after disease progression on ICI [71]. In the phase III randomized DREAMseq trial with 265 patients with treatment-naive BRAF-mutant melanoma, a -preliminary analysis revealed that, compared with the sequence of targeted therapy followed by immunotherapy, upfront immunotherapy followed by targeted therapy improved OS, with the 2-year OS rate increasing from 52 to 72% (log-rank p = 0.0095) [71]. This trial was stopped early because of this OS benefit.

Of the ICI-based regimens approved for metastatic and recurrent melanoma, the combination therapy with nivolumab and ipilimumab [72, 73] is preferred over single-agent pembrolizumab [74] or nivolumab [75] because of improved efficacy. The combination of nivolumab and relatlimab was also approved based on the RELATIVITY-047 trial [76]. Relatlimab is the first drug in the class approved to target another immune checkpoint, lymphocyte-activation gene 3 (LAG-3) expressed on effector T cells and regulatory T cells. It is associated with T-cell exhaustion and resistance to immunotherapies such as PD-1 blocking antibodies. This combination significantly improves PFS, but a non-statistically significant trend towards improved OS was observed.

Because of the high success of ICI in advanced melanoma, tremendous interest has been shifted to early-stage melanoma in adjuvant and neoadjuvant settings. Long before ICI was studied, immunotherapy with interferon was extensively studied both in advanced and localized melanoma. However, secondary to the efficacy and safety profiles, drug availability, and other factors, most clinicians recommend single-agent immunotherapy with nivolumab or pembrolizumab. Nevertheless, it is anticipated that combination immunotherapy will be examined in the neoadjuvant setting for high-risk localized melanoma.

#### Adjuvant interferon alfa-2b

Interferon alfa-2b now mainly remains a historical adjuvant therapy after more effective and less toxic ICIs have been approved. Based on the Eastern Cooperative Oncology Group 1684 and 1694 trials as well as meta-analysis of trials with various doses and schedules, interferon alfa-2b improves OS compared to the control [77–80]. However, interferon is associated with significant unpleasant side effects, such as flu-like symptoms and depression. As discussed above, the intergroup E1609 trial [81] has shown that ICIs are more effective than interferon. Furthermore, the manufacturer has discontinued the production of interferon alfa-2b. Currently, there is almost no role of interferon alfa-2b as a therapeutic option in any malignancy, including melanoma.

#### Adjuvant nivolumab

In the CheckMate 238 trial, 906 patients with stage IIIB, IIIC or IV melanoma who had complete resection of melanoma were randomized to receive one year of adjuvant nivolumab or ipilimumab (10 mg/kg every three weeks for four cycles followed by every 12 weeks for one year). At a median follow-up of 51 months, the 4-year recurrence-free survival was 51.7% with nivolumab compared with 41.2% with ipilimumab (HR 0.71; 95% CI 0.60–0.86; p = 0.0003) [82, 83]. Five-year OS was not significantly different: 76% versus 72% (HR 0.86; 95% CI 0.66–1.12). In the BRAF-wild-type group, nivolumab also improved five-year RFS (47% versus 36% with ipilimumab, HR 0.69; 95% CI 0.53–0.9). Among 42% (381) of patients with BRAF-mutant tumors, nivolumab adjuvant therapy trends towards improved five-year RFS: 50% versus 42% with ipilimumab (HR 0.8; 95% CI 0.6–1.05).

#### Adjuvant pembrolizumab

In the double-blind, placebo-controlled phase III European Organization for Research and Treatment of Cancer (EORTC) 1325/KEYNOTE-054 trial, 1019 patients with completely resected stage III melanoma were randomized to one year of adjuvant pembrolizumab or placebo [84–86]. With a median follow-up of 42 months, pembrolizumab significantly increased the 3.5-year relapse-free survival from 49.4% of the placebo group to 65.3% (HR 0.60; 95% CI 0.49–0.73; p < 0.0001). Similar results were observed with the coprimary endpoint of RFS in the 853 patients with PD-L1-positive tumors: 51.6% with the placebo versus 66.7% with pembrolizumab (HR 0.61; 95% CI 0.49–0.76; p < 0.0001).

#### Adjuvant ipilimumab

The phase III EORTC18071 trial that led to the U.S. FDA approval of ipilimumab as an adjuvant therapy at 10 mg/kg every three weeks for four doses followed by every three months for up to three years. In that trial, 951 patients were randomized to ipilimumab or placebo. With a median follow-up of 5.3 years, the 5-year recurrence-free survival was 40.8% with ipilimumab compared with 30.3% with placebo (HR 0.76; 95% CI 0.64–0.89; p < 0.001) [87, 88]. In a subsequent North American Intergroup E1609 trial, ipilimumab at 3 mg/kg, but not at 10 mg/kg, significantly improved OS (HR 0.78; 95.6% CI 0.61–0.99; p = 0.044) compared with interferon alfa-2b [81]. Hence, if ipilimumab is used as adjuvant therapy, a dose of 3 mg/kg is commonly used.

#### Adjuvant pembrolizumab for high-risk localized disease without lymph node involvement (stage IIB and IIC)

The benefit of ICIs has been further extended to high-risk resected melanoma without lymph node involvement. This group of patients mainly have melanoma with deep local disease (> 4 mm) or with relatively superficial disease (2–4 mm) with ulceration but without lymph node metastasis. In this scenario, adjuvant pembrolizumab is commonly recommended based on the KEYNOTE-716 trial, although surveillance and enrollment in a clinical trial are reasonable alternatives. In this double-blinded, placebo-controlled phase III trial, 976 patients with stage IIB and IIC melanoma were randomized to pembrolizumab every three weeks for 17 cycles or a placebo. PFS was the primary endpoint. At a median follow-up of 21 months, pembrolizumab therapy significantly prolonged PFS, with an 18-month PFS of 86% versus 77% in the placebo group (HR 0.61; 95% CI 0.45–0.82) [89].

#### ICI-based neoadjuvant therapy

Built on the success of adjuvant immunotherapy in melanoma, neoadjuvant ICI-based therapy is actively being explored. In the randomized Phase II SWOG 1801 trial, patients with Stage IIIB-IV cutaneous, acral or mucosal melanoma were randomized to surgery followed by pembrolizumab adjuvant therapy for 18 cycles (54 weeks, adjuvant arm) or neoadjuvant pembrolizumab for three cycles, followed by surgery and 15 cycles of adjuvant pembrolizumab (neoadjuvant arm). Patients in the neoadjuvant arm had significant improvement in event-free survival, the study primary endpoint (one-sided log-rank p = 0.0015, Cox HR 0.59, 95% CI 0.40–0.86) [90]. In addition, the neoadjuvant nivolumab and ipilimumab combination has also been explored as a neoadjuvant therapy for locally advanced melanoma with exciting efficacy in several Phase I and II trials [91–95].

### Gastrointestinal cancers

#### Gastric/gastroesophageal junction cancer

Gastric cancer, including gastroesophageal junction (GEJ) cancer, is the fourth leading cause of cancer-related deaths worldwide. Outcomes remain poor with standard-of-care fluoropyrimidine and platinum-based chemotherapy in unresectable diseases [96, 97]. Current ICIs approved as first-line agents for advanced or metastatic gastric/GEJ cancers include nivolumab in conjunction with chemotherapy (CheckMate 649) [98] and pembrolizumab in conjunction with chemotherapy and trastuzumab for HER2 + tumors (KEYNOTE811) [99].

##### Adjuvant nivolumab for gastric/GEJ cancer

Currently, the role of ICI as an adjuvant therapy has been substantiated for squamous cell esophageal cancer. Preoperative chemoradiation has been a standard of care for this patient population. Complete pathological response to preoperative chemoradiation is a well-established prognostic marker for superior outcomes following surgical resection, whereas residual diseases are associated with worse outcomes. Nivolumab demonstrated its significant clinical benefit in the adjuvant setting in the global randomized double-blind placebo-controlled phase III CheckMate 577 study of patients with stage II/III disease following definitive treatment with chemoradiation and surgical resection with residual disease [100]. In this trial, 794 patients with esophageal or gastroesophageal junction cancer and residual pathological disease at the time of surgery were randomized at a 2:1 ratio to adjuvant nivolumab for one year or placebo. At a median follow-up of 24.4 months, adjuvant therapy significantly improved the median disease-free survival, the primary endpoint, from 11.0 months of the placebo to 22.4 (HR 0.69; 95% CI 0.56–0.86; p < 0.001), and the benefits were seen across all patient subgroups. The overall survival data were premature.

##### Neoadjuvant immunotherapy for gastric/GEJ cancer

These encouraging results from the utilization of immunotherapy as the first-line therapy for adenocarcinoma of the stomach, esophagus, and GEJ have led to the development of immunotherapy as neoadjuvant therapy for this patient population. Approximately 30% of this patient population has resectable adenocarcinoma at the time of diagnosis; the five-year survival is estimated at 45% following local definitive therapy if it is diagnosed at an advanced clinical stage (cT2 or higher, nodal positive stage, or both). The current standard of care for this locally advanced resectable gastric cancer is the perioperative chemotherapy combination of fluorouracil /leucovorin, oxaliplatin, and docetaxel, which offers a median overall survival of 50 months [101]. Several completed or ongoing studies have examined the role of ICI in combination with chemoradiation in the preoperative setting, including three phase III studies, each testing one anti-PD-1 antibody in combination with chemoradiation compared with chemoradiation alone as neoadjuvant treatment. As neoadjuvant or perioperative systemic therapy has been a well-established paradigm for gastric and GEJ cancers, the future development of immunotherapy for resectable diseases is anticipated to continue to follow this pattern. For example, a phase III study (NCT04882241) randomized patients with gastric cancer to receive pembrolizumab plus chemotherapy or placebo plus chemotherapy as neoadjuvant and adjuvant treatments. Primary endpoints include event-free survival, pathological complete response rate, and OS.

### ***Colorectal cancer***

Colorectal cancer is the third leading cause of cancer death in both men and women in the U.S. and most parts of the world [102]. Currently, all ICIs proven to be effective for treating colorectal cancers have been limited to metastatic colorectal cancers (mCRCs) with MSI-H/dMMR (high levels of microsatellite instability/deficient mismatch repair) [103]. In 2017, the U.S. FDA approved pembrolizumab as a single agent for patients with MSI-H/dMMR mCRC that progressed following treatment with fluoropyrimidine, oxaliplatin, and irinotecan. Since then, multiple ICIs have demonstrated their efficacy in mCRC [104]. Pembrolizumab subsequently demonstrated superior median PFS (16.5 vs. 8.2 months) and OS (13.7 vs. 10.8 months) compared with 5-fluorouracil-based therapy ± bevacizumab or cetuximab as first-line therapy for unresectable/metastatic MSI-H/dMMR mCRC in the KEYNOTE-177 study [105]. The durable response of these ICIs for mCRC patients with the MSI-H/dMMR subtype highlights the potential of immunotherapy as adjuvant therapy for this patient population. Currently, a phase III study is ongoing to compare the anti-PD-L1 antibody atezolizumab in combination with chemotherapy with chemotherapy alone in MSI-H CRC (clinicaltrials.gov identifier No: NCT02997228).

In addition, there is significant interest in testing ICIs as neoadjuvant therapy in combination with either radiation alone or with chemoradiation for MSI-H rectal cancer. Such efforts may lead to a paradigm change as patients with a complete clinical response to neoadjuvant therapy have been selected for watchful waiting without surgical resection. If immunotherapy or a combination of immunotherapy with chemoradiation leads to a higher complete response rate in MSI-H rectal cancer, more patients would be spared from morbid surgical resection. Strikingly, a recent study of 14 patients with locally advanced MSI-H rectal cancer reported that all 14 patients had a complete response to a six-month course of neoadjuvant anti-PD-1 antibody treatment, sparing them not only from surgical resection but also chemotherapy and radiotherapy [106].

However, ICI for the majority of CRC patients without MSI-H/dMMR does not seem to be beneficial [107].

### ***Hepatobiliary cancer***

Hepatocellular carcinoma (HCC) is considered relatively chemorefractory, and conventional cytotoxic chemotherapy has become less popular in treating HCC. Since 2008, targeted therapy and immunotherapy have gained increasing popularity, but with limited efficacy. ICI-based combination therapies are now the first-line treatment for locally advanced and metastatic HCC [108], and tyrosine kinase inhibitors are the second-line treatment. Immunotherapy should be avoided in patients after allogeneic liver transplantation, as it can trigger anti-allograft immune rejection.

For advanced HCC with a Child‒Pugh class A on the liver function scale, the combination of atezolizumab and bevacizumab was approved by the U.S. FDA, based on the Phase III IMBrave 150 trial [108]. At a median follow-up of 15.6 months, the atezolizumab and bevacizumab combination significantly improved the OS from 13.4 months in the sorafenib control group to 19.2 months (HR 0.66; 95% CI 0.52–0.85; p < 0.001), PFS from 4.3 months to 6.9 months (HR 0.65; 95% CI 0.53–0.81; p < 0.001), and objective response rate from 11 to 30% (p < 0.001) [109]. In addition, the durvalumab plus an anti-CTLA4 antibody, tremelimumab, combination was first tested in a phase I/II trial [110]. A subsequent phase III HIMALAYA trial showed that the primary endpoint of OS was met, with a median OS of 16.4 months with the tremelimumab/durvalumab combination compared with 13.8 months (HR, 0.78; 96% CI; 0.65–0.92; p = 0.0035) and an objective response rate of 20.1% versus 5.1% [111].

For localized HCC with sufficient liver function, surgical resection with curative intent is commonly performed, and no other neoadjuvant or adjuvant therapies are widely accepted. Adjuvant antiviral therapy improves treatment outcomes for hepatitis B-related HCC and is recommended for those patients with an active viral infection. Chemotherapy has failed to demonstrate a significant role as adjuvant therapy for localized HCC. Targeted therapy primarily has a cytostatic antitumor effect and is hence unlikely to be an effective adjuvant therapy. The long-lasting effect of ICI has been observed in clinical trials, thus making ICI an ideal adjuvant therapy in HCC. At the 2021 China Cancer Immunotherapy Workshop, Richard Finn, MD, from the University of California, Los Angeles, provided updates on the recent developments in immunotherapy for HCC. The phase III KEYNOTE-937 (NCT03867084) clinical trial randomized 950 patients to compare adjuvant pembrolizumab with placebo in Child‒Pugh A HCC with a complete radiographic response after surgery or local ablation. Recurrence-free survival (RFS) and OS were the primary endpoints [112]. A few other trials are also going on, including the Checkmate 9DX and IMbrave 050 trials [113]. A study with neoadjuvant immunotherapy for resectable HCC is currently being designed to evaluate the biological response to immunotherapy.

### Head and neck squamous cell cancer (HNSCC)

HNSCC includes cancers of the oral cavity, oropharynx, nasopharynx, hypopharynx, and larynx. For early-stage, localized and locoregionally advanced HNSCC, the treatment intent is curative. For locally advanced HNSCC, combined therapeutic modalities are commonly adopted to achieve better control, improved function preservation, superior long-term survival and can be curative in a small percentage of patients. Depending on the tumor size and location, the patient’s general health and comorbidities, and local expertise, patients can undergo surgery followed by postoperative radiation with or without chemotherapy, induction chemotherapy followed by surgery or radiation, or sequential or concurrent chemoradiotherapy without surgery. Surgery is the main therapy for oral cavity cancer supplemented with radiation with or without chemotherapy. For large cancers at other locations, function-preserving approaches, mainly radiation-based combined therapeutic modalities, are adopted.

Curative treatments, including surgery and chemoradiation, can result in high morbidity. ICIs are tested in the neoadjuvant setting in an attempt to downstage the disease for a less morbid surgical resection. Multiple phase II neoadjuvant studies have been completed, and most trials have demonstrated a better pathological treatment response (pTR) with ICI treatment as well as the association of pTR with better survival outcomes following surgery. Uppaluri et al. (2020), reported a single-arm study of 36 patients with locally advanced, resectable HNSCC, most of whom had oral cavity cancers [114]. There were no significant surgical delays and no grade 3 or 4 adverse events after one cycle of pembrolizumab followed by surgery 2–3 weeks later. Pathologic tumor response occurred in 44% (16/36) of the patients. Another study reported neoadjuvant and adjuvant nivolumab combined with the NK-cell checkpoint inhibitor lirilumab in patients with recurrent resectable HNSCC [115]. Among the 28 patients, there were no delays in surgery, and grade 3–4 adverse events occurred in 11%. While 96% showed stable disease at the time of surgery, a pathological response was observed in 43% (12/28) of the patients. In another phase II trial with 92 patients, neoadjuvant pembrolizumab showed a 97% one-year DFS in the intermediate-risk group and 66% in the high-risk group [116]. Encouraged by promising results from phase II studies, multiple phase III studies have been initiated with the results still pending.

### Gynecological malignancies

Immunotherapy has been approved for several gynecological malignancies, a highly diversified disease group, mostly for recurrent or advanced disease. Endometrial carcinoma is the second most common malignancy associated with MSI-H or dMMR after colorectal cancer. The anti-PD-1 antibody dostarlimab is now approved by the U.S. FDA as a second-line therapy, following platinum-based chemotherapy, for patients with dMMR/MSI-H endometrial cancers [117].

For recurrent and metastatic cervical cancer, the activity of immunotherapy was demonstrated in the Phase III KEYNOTE-826 trial. Compared with chemotherapy with or without bevacizumab, the addition of pembrolizumab as the first-line treatment significantly improved PFS from 8.2 months to 10.4 months (HR 0.62; 95% CI 0.50–0.77; p < 0.001) and 2-year OS from 41.7% to 53.0% (HR 0.64; 95% CI 0.50–0.81; p < 0.001) [118]. Based on this trial, the U.S. FDA approved pembrolizumab in combination with platinum-based chemotherapy, with or without bevacizumab, for the treatment of patients with persistent, recurrent or metastatic cervical cancer with PD-L1 CPS ≥ 1.

Direct evidence supporting the role of ICIs for treating cervical and endometrial cancers in the adjuvant or neoadjuvant setting is still lacking, but there is a strong rationale for testing ICIs in clinical trials. Currently, four phase II clinical trials of neoadjuvant therapy with an anti-PD-1 antibody in combination with paclitaxel and platinum-based chemotherapy for locally advanced cervical cancer (NCT04516616, NCT0423898, NCT05013268, NCT04799639), one clinical trial of adjuvant therapy with anti-PD-1 antibody sintilimab in combination with the standard of care chemoradiation for resected cervical cancer (NCT04918628), and one clinical trial of adjuvant therapy with pembrolizumab plus chemotherapy with or without radiation for resected endometrial cancer (NCT04634877) are ongoing.

Ovarian cancer is relatively sensitive to chemotherapy and resists to ICI treatment. The role of immunotherapy in metastatic ovarian cancer still needs to be determined.

#### Concluding remarks

Immunotherapy with ICIs has revolutionized the treatment of various cancers, initially for more advanced disease and, more recently, for early-stage tumors. With numerous ongoing clinical trials, we expect to see more immunotherapy approvals for early-stage cancers. Similar to those with advanced cancer, only a minority of patients benefit from ICIs as a monotherapy. One approach to counteract this predicament is the biomarker-based selection of patients who are more likely to respond to ICIs, as seen in lung cancer with high PD-L1 expression, cancers with mismatch repair deficiency or cases with a high tumor mutation burden [10, 119, 120]. However, this population is small among all cancer patients. We believe the future of cancer immunotherapy will be combination therapy that induces multiple hits on cancers and achieves synergistic effects [8, 121]. To date, the combinations of two ICIs, ICIs with chemotherapy and ICIs with targeted therapy, have already obtained U.S. FDA approvals, and more combinations, particularly with chimeric antigen receptor (CAR)-engineered immune cells, are being explored.

## Data Availability

The material supporting the conclusion of this review has been included within the article.

## Abbreviations

5-FU: Fluorouracil
ABCP: Atezolizumab plus bevacizumab
ACP: Atezolizumab
ADC: Antibody‒drug conjugates
AJCC: American joint committee on cancer
ALK: Anaplastic lymphoma kinase
BCG: Bacillus calmette-guerin
BCLC: Barcelona clinic liver cancer
BCP: Bevacizumab
BCT: Breast-conserving therapy
BICa: Urothelial bladder cancer
BRCA: Breast cancer gene
CAHON: Chinese American Hematologist and Oncologist Network
CDK: Cyclin-dependent kinase
CI: Confidence interval
CPS: Combined positive score
CR: Complete response
CTLA-4: Cytotoxic T-lymphocyte-associated protein 4
DCR: Disease control rate
DFS: Duration-free survival
dMMR: Mismatch repair protein deficient
EGFR: Epidermal growth factor receptor
EORTC: European organization for research and treatment of cancer
ES: Extensive-stage
ET: Endocrine therapy
FDA: Food and drug administration
GEJ: Gastroesophageal junction
GI: Gastrointestinal
HAIC: Hepatic arterial infusion chemotherapy
HCC: Hepatobiliary carcinoma
HER2: Human epidermal growth receptor 2
HNSCC: Head and neck squamous cell carcinomas
HPV: Human papillomavirus
HR: Hazard ratio
ICI: Immune checkpoint inhibitor
IL-2: Interleukin-2
IO: Immuno-oncology
LAG-3: Lymphocyte-activation gene 3
LS: Limited-stage
mCRC: Metastatic colorectal cancer
MEK: Mitogen-activated protein kinase kinase
MRI: Magnetic resonance imaging
MSI-H: Microsatellite instability-high
MSI-L: Microsatellite instability-low
NMIBC: Non-muscle-invasive bladder cancer
NMPA: National medical product administration
NSCLC: Non-small cell lung cancer
ORR: Objective response rate
OS: Overall survival
PARP: Poly adenosine diphosphate-ribose polymerase
PCI: Prophylactic cranial irradiation
pCR: Pathological complete response
PD-1: Programmed cell death protein 1
PD-L1: Programmed death-ligand 1
PFS: Progression-free survival
pMMR: Proficient mismatch repair
pTR: Pathological treatment response
RCC: Renal cell carcinoma
RECIST: Response evaluation criteria in solid tumors
RFS: Recurrence-free survival
RT: Radiation therapy
SCLC: Small cell lung cancer
SLNB: Sentinel lymph node biopsy
TACE: Trans-arterial chemoembolisation
T-DM1: Ado-trastuzumab emtansine
TNBC: Triple-negative breast cancer
TNM: Tumor, Node, Metastasis
TURBT: Transurethral resection of bladder tumor
UICC: Union for international cancer control
vs.: Versus
